# New Insights into the p38**γ** and p38**δ** MAPK Pathways

**DOI:** 10.1155/2012/520289

**Published:** 2011-11-30

**Authors:** Ana Risco, Ana Cuenda

**Affiliations:** Departamento de Inmunología y Oncología, Centro Nacional de Biotecnología-CSIC, Campus de Cantoblanco-UAM, 28049 Madrid, Spain

## Abstract

The mammalian p38 mitogen-activated protein kinases (MAPKs) family is composed of four members (p38**α**, p38**β**, p38**γ**, and p38**δ**), which are very similar in amino acid sequence but differ in their expression patterns. This suggests that they may have specific functions in different organs. In the last years most of the effort has been centred on the study of the function of the p38**α** isoform, which is widely referred to as p38 in the literature. However, the role that other p38 isoforms play in cellular functions and their implication in some of the pathological conditions have not been precisely defined so far. In this paper we highlight recent advances made in defining the functions of the two less studied alternative p38MAPKs, p38**γ** and p38**δ**. We describe that these p38MAPKs show similarities to the classical p38**α** isoform, although they may play central and distinct role in certain physiological and pathological processes.

## 1. Introduction

To preserve the homeostasis and health of the organism cells are constantly responding to changes in the physical and chemical properties of the environment by altering many of their cellular functions. The activation of Mitogen Activated Protein Kinases (MAPKs) is involved in the transduction of most extracellular signals, and it is one of the major signal transduction mechanism by which the cell adapts to changes in the surrounding medium. In mammalian cells there are four well-characterised MAPK families: ERK1/2, ERK5, JNK, and p38MAPK, which are serine/threonine kinases that catalyze the reversible phosphorylation of proteins.

The p38MAPK family comprises four members, p38*α*, p38*β*, p38*γ*, and p38*δ*. The isoform p38*α* was identified in 1994 by four groups as a 38 kDa polypeptide that is activated in response to endotoxin treatment, cell stress, or cytokines [1]. Two to three years later, three additional isoforms were described: p38*β* [2–4], p38*γ* [5, 6], and p38*δ* [7, 8]. These kinases share highly similar protein sequences; p38*α* and p38*β* are 75% identical, whereas p38*γ* and p38*δ* are 62% and 61% identical to p38*α*, respectively. In turn, p38*γ*, and p38*δ* are ~70% identical to each other. The four p38MAPK isoforms are widely expressed, although p38*β*, p38*γ* and p38*δ* expression appear to be higher in specific tissues; for example, p38*β* is abundant in brain, p38*γ* in skeletal muscle, and p38*δ* in endocrine glands [1, 9]. In general, all p38MAPKs are strongly activated by a wide variety of environmental and cellular stresses or by inflammatory cytokines and are poorly activated by serum or growth factors [1]. The p38MAPK family can be further divided into two subsets based on sequence homology, substrate specificities, and sensitivity to chemical inhibitors, with p38*α* and p38*β* in one group and p38*γ* and p38*δ* in the other. In this paper, we provide an overview of the less known p38MAPK isoforms, the p38*γ* and p38*δ* MAPK pathways, which are strongly activated by stress, but also play important roles in tissue regeneration, differentiation, metabolic diseases, and cancer.

## 2. General Features of p38**γ** and p38**δ** MAPKs

Human p38*γ* and p38*δ* isoforms are serine/threonine protein kinase of 367 and 365 amino acids with a predicted molecular mass of 42–45 kDa and are encoded by different genes located on chromosomes 22q13.3 and 6p21.31, respectively [1, 7, 10]. p38*γ* is also known as ERK6, SAPK3, and MAPK12. It was first described by three independent studies as either a MAPK involved in myoblast differentiation [5], a stress-activated protein kinase (SAPK) highly expressed in skeletal muscle [6], or a new member of the p38MAPK family [11]. p38*δ*, also known as SAPK4 and MAPK13, was cloned as the fourth member of the p38MAPK family by two different groups [7, 8].

The structure of doubly phosphorylated, active p38*γ* in complex with an ATP analog has been determined by X-ray crystallography [12]. The global structure of p38*γ* is similar to other enzymes of the MAPK family and is characterized by two domains separated by a deep channel where potential substrates might bind. The dually phosphorylated p38MAPK goes through characteristic global conformational changes that alters the alignment of the two kinase halves (N-terminal and C-terminal domains) of the folded protein and enhances access to substrate. In addition, the interaction between MAPKs and their upstream activators seems to work allosterically to make the MAPKs activation loop available for processing by kinases and phosphatases, which further increases enzymatic activity [12–14]. Although the conformation of p38*γ* activation loop is almost identical to that observed in the structure of activated ERK2, contrary to ERK2, the crystal structure of activated p38*γ* exists as a monomer, suggesting that not all activated MAPKs form dimers [12]. A feature that makes p38*γ* unique among other MAPKs is its short C-terminal sequence-KETXL, an amino acid sequence which docks directly to PDZ domains of proteins, such as *α*1-syntrophin, SAP (synapse-associated protein) 90/PSD (postsynaptic density) 95 and SAP97/hDlg (human disc large), and phosphorylation of these proteins by p38*γ* is dependent on its binding to the PDZ domains [15–17].

The information about p38*γ* and p38*δ* biological role is limited compared to the extensive knowledge of p38*α* and p38*β* functions. This is at least in part due to the lack of specific inhibitors for p38*γ* and p38*δ*. *In vitro* and *in vivo* assays demonstrated that only p38*α* and p38*β* are inhibited by certain compounds, such as SB203580 and other pyridinyl imidazoles, whereas p38*γ* and p38*δ* are completely unaffected by these drugs [7, 18, 19]. This is mainly due to the differences, between p38*γ* and p38*δ* compared to p38*α* and p38*β*, in the amino acid sequence of the ATP-binding pocket, the site where most protein kinase inhibitors bind and directly compete with ATP [1].

## 3. Regulation of p38**γ** and p38**δ**

**Figure 1:**
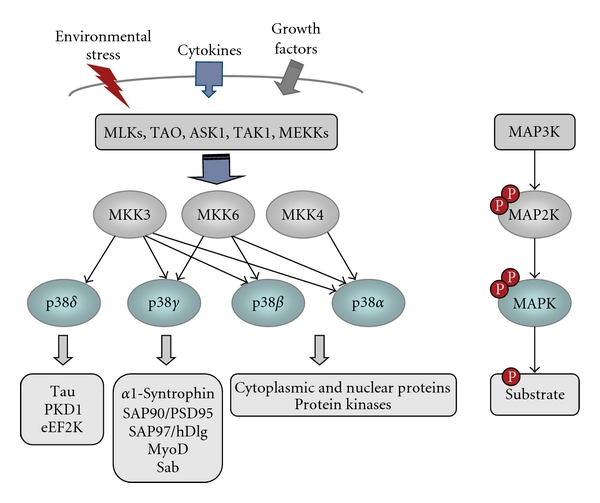
The p38MAPK pathway. p38*γ* and p38*δ* MAPK substrates identified so far are shown.

The canonical activation of p38MAPKs occurs via dual phosphorylation of tyrosine and threonine residues in a conserved TGY motif, located in the activation loop of kinase subdomain VIII. MAPK phosphatases reverse this phosphorylation and return the p38MAPK to their inactive state. Phosphorylation of p38MAPKs is catalysed by the dual specificity kinases (MKK or MAP2Ks), MKK3 and MKK6, which are in turn activated upon phosphorylation of serine/threonine residues by phosphorylation by a MAPK kinase kinase (MAP3K) (Figure 1). The MAP3K responsible for activating the p38MAPK pathways appears to be cell type and stimulus specific. Several MAP3Ks have been implicated in the regulation of p38MAPK signalling, these include MLKs (mixed-lineage kinases), ASK1 (apoptosis signal-regulating kinase-1), TAO (thousand and one amino acid) 1 and 2, TAK1 (TGF *β*-activated kinase 1), and some members of the MEKK (MAPK/ERK kinase kinase) family [20]. The diversity of MAP3Ks and their ability to activate also other MAPKs provide a mechanism to respond to many stimuli and to integrate different signalling pathways. It has been shown that MAP3K of the p38MAPK pathway are regulated by binding to low molecular weight GTP-binding proteins, ubiquitination or phosphorylation by STE20 family members [1, 20].

MKK3 and MKK6 are highly selective for p38MAPKs and do not activate other MAPKs [1]. The major MKK required for the activation of specific p38MAPK may be determined by several factors: one is the cell type as the level of expression varies [21, 22]; another is the nature and also the strength of the stimuli. Since MKK6 can activate all p38 isoforms *in vitro*, it has been suggested that the pattern of downstream p38MAPK activation in the particular response may be determined by the level of MKK6 activity triggered by a given stimulus [23]. Moreover, there are two important structural requirements for selective activation of p38MAPK isoforms by MKKs: docking sequences in the N-terminus of the MKK and isoform-specific sequences of the p38MAPK isoforms within the activation loop [13, 24, 25]. Using MKK-targeted gene disruption and small interfering RNA (siRNA) approaches, it has been shown that, in response to most stimuli, MKK3 and MKK6 are the main p38*α* activators but, in some circumstances, such as ultraviolet radiation, MKK4, an activator of JNK, may contribute to p38*α* activation [26]. Moreover, although it has been shown that *in vitro* experiments MKK4 also phosphorylates and activates p38*γ* and p38*δ* [7, 27], studies utilizing mouse embryonic fibroblasts lacking MKK3 and/or MKK6 indicate that activation of distinct p38MAPK isoforms is regulated by the selective and synchronized action of the two MKKs, in response to cell stress. Thus, both MKK3 and MKK6 are essential for p38*γ* activation induced by environmental stresses, whereas MKK6 is the major p38*γ* activator in response to the cytokine tumour necrosis factor-*α* (TNF*α*). On the other hand, MKK3 is the major kinase responsible for p38*δ* activation by ultraviolet radiation, hyperosmotic shock, TNF*α* or by the protein synthesis inhibitor anisomycin (Figure 1) [17]. Supporting this is the finding that endogenous p38*δ* activation in response to TGF*β*1 is impaired in glomerular mesangial cells from MKK3-deficient mice [22]. Nonetheless, the relative contribution of MKK3 and MKK6 to p38*γ* and p38*δ* activation might strongly depend not only on the nature and strength of the stimulus, but also on the cell type.

The magnitude and duration of p38MAPK signal transduction are critical determinants of its biological effects. Termination of p38 kinase catalytic activity involves the activity of several phosphatases that target the activation loop threonine and tyrosine residues. In mammalian cells there are good *in vivo* pieces of evidence for p38*α* activity downregulation by several protein phosphatases, including protein serine/threonine phosphatases (PPs) [28, 29], protein tyrosine phosphatases (PTPs) [30], and dual-specificity phosphatases (DUSPs, also known as MAPK phosphatases (MKPs)) [31]. However, their role in p38*γ* and p38*δ* dephosphorylation has not been extensively studied, and therefore very little is known about physiological p38*γ* and p38*δ* protein phosphatases. Recently, it has been shown in one study that p38*γ* interacts through its C-terminal binding PDZ motif with the single PDZ domain of the protein tyrosine phosphatase PTPH1. Moreover, PTPH1 can dephosphorylate p38*γ*, but not p38*α*, *in vitro* and in overexpression experiments in cells. This specificity seems to be determined by both p38*γ* C-terminal PDZ-binding sequence and the conserved TGY motif within the kinase subdomain [32].

## 4. p38**γ** and p38**δ** Substrates and Biological Functions

p38MAPK family members have overlapping substrate specificities, and the genetic ablation of specific p38MAPK family members has also demonstrated the existence of functional redundancy [16]. However, there are some differences, with particular substrates being better phosphorylated by p38*α* and p38*β* than p38*γ* and p38*δ* or vice versa. For example, MAPK-activated protein kinase 2 (MK2) and MK3 are very good substrates for p38*α* and p38*β*, but cannot be phosphorylated by other p38MAPK isoforms [1].

The lack of specific inhibitors for p38*γ* and/or p38*δ* has slowed down the identification of their *in vivo* substrates and the elucidation of their biological roles. Nonetheless, this problem can be partly solved by the use of p38 knockout mouse models. p38*γ* and p38*δ* and double p38*γ*/p38*δ* knock-out mice have been generated, which are viable and fertile [16]. Moreover, the diaryl urea compound BIRB796 is not only a potent inhibitor of p38*α* and p38*β*, but also inhibits p38*γ* and p38*δ* at higher concentrations in cell-based assays providing a new tool for identifying physiological roles of these two p38MAPK isoforms [19, 33].

**Figure 2:**
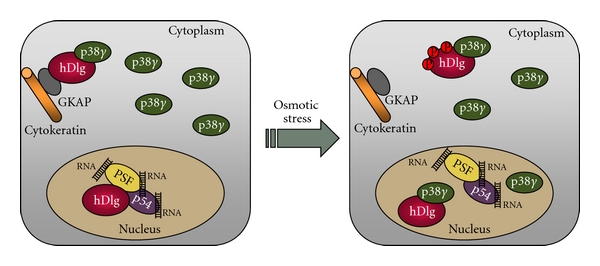
The involvement of p38*γ* in the regulation of nuclear and cytoplasmic protein complexes. In the nucleus of resting cells SAP97/hDlg complexes with PSF/p54-RNAs, whereas in the cytoplasm it interacts at the cytoskeleton with both the protein GKAP and a fraction of p38*γ*, which is localized mainly in the cytoplasm. Changes in the osmolarity of the environment causes: (i) p38*γ* activation in the cytoplasm, which phosphorylates SAP97/hDlg causing its dissociation from GKAP and therefore from the cytoskeleton, (ii) accumulation of p38*γ* in the nucleus, and (iii) the nuclear interaction of p38*γ* with SAP97/hDlg, which leads to its dissociation from PSF/p54-RNAs independently of SAP97/hDlg phosphorylation.

Several physiological substrates for p38*γ* MAPK isoform have been described in the past years (Figure 1). A feature that makes p38*γ* unique among all MAPKs is its short C-terminal sequence ideal for binding PDZ domains in proteins. p38*γ* binds to the PDZ domain of a variety of these proteins, such as *α*1-syntrophin, SAP90/PSD95, and SAP97/hDlg, and under stress conditions is able to phosphorylate them and modulate their activity [15–17]. One valuable tool used in the identification of p38*γ* substrates has been the cell permeant peptide TatSAPK3C which contains the last nine residues of p38*γ* fused to the cell-membrane transduction domain of the human immunodeficiency virus-type 1 (HIV-1) Tat protein. This peptide blocks the phosphorylation of PDZ domain-containing proteins by p38*γ* in intact cells by preventing the association of the kinase with the PDZ domain of the substrate [16, 17]. These PDZ domain-containing proteins are scaffold proteins usually targeted to the plasma membrane cytoskeleton at specialised sites such as the neuromuscular junction and gap junctions through protein-protein interactions. In the case of SAP97/hDlg its phosphorylation by p38*γ* provides a mechanism of dissociating it from the cytoskeleton [16], which indicates a role of this p38MAPK isoform in modulation of cytoskeletal organization. SAP97/hDlg is the mammalian homologue of the *Drosophila* tumour suppressor Dlg, a scaffold protein that forms multiprotein complexes with a variety of proteins and is targeted to the cytoskeleton by its association with guanylate kinase-associated protein (GKAP). The p38*γ*-catalysed phosphorylation of SAP97/hDlg triggers its dissociation from GKAP and therefore releases it from the cytoskeleton (Figure 2). This is likely to regulate the integrity of intercellular complexes, cell shape, and volume as an adaptive mechanism to changes in the environment [16].

SAP97/hDlg also localizes in the nucleus where it forms a complex with the proteins polypyrimidine tract-binding (PTB) protein-associated splicing factor (PSF) and p54^nrb^, and with various RNAs, [34]. PSF and p54^nrb^ are nucleic acid-binding proteins that associate *in vivo* and regulate transcription, pre-mRNA processing, nuclear retention of defective RNA, as well as DNA unwinding and repair [35]. p38*γ* regulates hDlg-PSF complex dissociation in the nucleus independently of hDlg phosphorylation by displacing PSF from hDlg, since both proteins, p38*γ* and PSF, bind to PDZ1 of hDlg [34]. p38*γ* accumulates in the nucleus after hyperosmotic stress (Figure 2), but not following other p38*γ*-activating stimuli such as UV irradiation. This indicates that the nature of the stimulus determines p38*γ* distribution and that some signals could release p38*γ* from docking molecules that retain it in the cytosol. Moreover, the nuclear accumulation of p38*γ* might be a response mechanism to some stimuli facilitating phosphorylation of p38*γ* targets in the nucleus. A nuclear role for p38*γ*, including functional interaction with SAP97/hDlg, would not exclude its distinct cytoplasmic role in modulating the SAP97/hDlg-cytoskeleton complex. Indeed, through its ability to shuttle between cytoplasm and nucleus, p38*γ*-SAP97/hDlg might provide a connection between two processes critical for adaptation to environmental changes: gene expression and cytoskeletal reorganization.

p38*γ* regulation of the SAP97/hDlg-PSF complex is independent of its kinase activity. This has been shown using cells from knockin mice expressing an endogenous inactive p38*γ* mutant in combination with cells from mice lacking p38*γ* [34]. Similarly, experiments in rat intestinal epithelial cells also suggest a phosphorylation-independent role for p38*γ* in K-Ras transformation, although the precise mechanism for this regulation remains unknown [36]. p38MAPKs act normally by direct phosphorylation of substrates on serine or threonine residues followed by proline. However, there are few examples showing that mammalian p38*α*- and yeast p38MAPK-related proteins such as Spc1 or Hog1 may also have kinase-independent roles (reviewed in [9]). Like p38*α*, p38*γ* seems to have a kinase-independent function by associating to protein targets and modulating their function in the absence of phosphorylation.

p38*γ* substrates that do not require PDZ domain-binding interactions are the mitochondrial protein Sab [37] and the transcription factor MyoD, whose phosphorylation by p38*γ* results in a decrease in its transcriptional activity [38].

Some p38*δ* substrates are proteins involved in the regulation of microtubule dynamics, suggesting that this p38MAPK may play a role in cytoskeletal remodelling. Thus, the protein stathmin and the neuronal microtubule-associated protein Tau are phosphorylated by p38*δ* *in vitro* and in transfected cells [39–42]. Tau function is modulated by phosphorylation, and its ability to bind and stabilise microtubules correlates inversely with its phosphorylation which may facilitate its self-assembly. Tau is a good *in vitro *p38*δ* and p38*γ* substrate, and its phosphorylation by these two kinases results in a reduction in its ability to promote microtubule assembly [39, 40]. Using a siRNA approach, p38*δ* has been reported to be the major Tau kinase in neuroblastoma in response to osmotic shock. p38*δ* phosphorylates endogenous Tau at residue threonine-50 (Tau-T50), which is phosphorylated in filamentous Tau from Alzheimer's disease brain. It seems that Tau-T50 phosphorylation is an early event after p38*δ* activation. Surprisingly, this phosphorilation causes an increase in the ability of Tau to promote microtubule assembly and help to the adaptive response of neurons to osmotic shock, whereas subsequent Tau phosphorylation at additional sites by p38*δ* or/and by other protein kinase(s) may then instead induce the detachment of Tau from the microtubule and destabilize the microtubule network [39].

Finally, it has been shown that p38*δ* phosphorylates and inactivates the eukaryotic elongation factor 2 (eEF2) kinase and the protein kinase D1 (PDK1) [43–45]. PDK1 controls insulin exocytosis in pancreatic beta cells, which suggests that p38*δ* plays a role in the regulation of insulin secretion [45].

## 5. Physiological Roles of p38**γ** and p38**δ** MAPK Pathways

Evidence from a number of studies carried out during the past few years suggests that many physiological functions of the p38MAPK isoforms may overlap but may not necessarily be redundant and/or identical [10]. Thus, during the last few years, studies using knock-out mice have provided important information concerning p38*γ*- and p38*δ*-functions *in vivo*. Contrary to p38*α*, whose constitutive deletion causes death during embryonic development [46–48], p38*γ* and p38*δ* deficient mice are viable and have not apparent phenotype [16]. Functional redundancy of all four p38MAPKs may contribute, at least in part, to the lack of evident phenotype of p38*γ*- and p38*δ*-deficient mice. Nonetheless, there are recent reports showing the implication of p38*γ* and p38*δ* in tissue regeneration, cancer, and metabolic diseases, further strengthening the interest of these pathways for the development of new therapeutics strategies. Thus, p38*δ* seems to be a regulator of processes related to the pathogenesis of diabetes, such as insulin secretion and *β* cells death. p38*δ*-deficient mice have improved glucose tolerance as a result of enhanced insulin exocytosis by pancreatic *β* cells. Correlating with this, p38*δ*-deficient mice show higher levels of active PKD1, which is known to positively regulate secretion in neuroendocrine cells, as a result of the lack of p38*δ*-mediated inhibitory phosphorylation. In addition, p38*δ* has been suggested as a potential therapeutic target for human diabetes, since p38*δ*-deficient mice are protected against the insulin resistance induced by a high-fat diet and the oxidative stress-mediated *β*-cell failure [45].

Using mainly ectopic expression and knock-down model cell lines it has been shown that p38*γ* and p38*δ* pathway could be involved in the modulation of some processes implicated in cellular malignant transformation, such as proliferation, cell cycle progression, or apoptosis [1, 10, 49, 50] indicating a potential oncogenic role of these kinases in cancer development and progression. In one study, p38*δ* promotes the malignant phenotype of squamous cell carcinoma by regulating cell proliferation and invasion [51]. In rat intestinal epithelial cells (IECs) and in human breast cancer, p38*γ* RNA and protein expression increases during Ras-induced transformation [36, 52]. p38*γ* knock-down in IEC blocks the Ras transformation activity and results in the significant diminution of the oncogenic characteristics of breast cancer cells [53–55]. Additionally, one recent study shows that p38*γ* mediates Ras-induced senescence at least partly by stimulating the transcriptional activity of p53 through direct phosphorylation; in contrast p38*α* appears to regulate senescence in a p53-independent manner [56]. These results indicate that increased p38*γ* gene expression is required for Ras oncogene activity but the mechanism by which p38*γ* may promote Ras transformation is not clear. Interestingly, p38*δ* was recently shown to mediate 12-O-tetradecanoylphorbol-13-acetate- (TPA-) induced epidermal cell proliferation in mice, and mice lacking p38*δ* show reduced susceptibility to the development of TPA-induced skin carcinomas [57]. All these results indicate the oncogenic function of p38*γ* and p38*δ*. Contrary, there is one study that shows pieces of evidence indicating that p38*γ* and p38*δ* have a role in the suppression of tumor development using mouse embryonic fibroblasts derived from mice lacking p38*γ* or p38*δ* [58]. Lack of either p38*γ* or p38*δ* increases cell migration and metalloproteinase-2 secretion, whereas only p38*δ* deficiency impairs cell contact inhibition. In addition, lack of p38*γ* in K-Ras-transformed fibroblasts leads to increased cell proliferation as well as tumorigenesis both *in vitro* and *in vivo* [58]. These discrepancies between different studies could be due not only to the difference in the experimental model and approaches used, but also to the distinct nature of cell(s) and process(es) that is/are involved in each experimental approach. Opposing roles in tumor development have also been reported for the isoform p38*α* [1, 59].

p38*δ* has been suggested to play an important role in inducing keratinocyte differentiation by regulating the expression of involucrin, which is a protein expressed during keratinocyte differentiation [60]. p38*δ* expression is detected in mouse and human epidermis [57, 61]. It has been shown that activation of exogenously expressed p38*δ* by differentiation-inducing agents correlates with increased involucrin promoter activity in keratinocytes [61, 62]. This occurs in a p38*α*/*β*-independent manner, and what is more, p38*γ* is poorly expressed in keratinocytes [63]. More data supporting the idea that p38*δ* may play a role in keratinocyte differentiation come from a study carried out in lesional psoriasis skin. Psoriasis is a chronic inflammatory skin disorder characterised by keratinocytes hyperproliferation and differentiation. It has been shown that the activity of p38*α*, p38*β*, and p38*δ* is augmented in lesional psoriasis skin compared with nonlesional psoriasis skin [64]. Additionally, p38*δ* may have a dual role in keratinocytes contributing not only to the differentiation process, but also to their apoptosis in a PKC*δ*-dependent manner, though the exact mechanisms by which p38*δ* may regulate keratinocyte differentiation or apoptosis are still unknown [65, 66]. It is important to notice that most of the pieces of evidence involving p38*δ* in regulating keratinocyte differentiation or apoptosis are based in overexpression experiments and require verification using other tools to both inhibit the activity or the expression of different p38MAPKs.

A possible p38*δ* and p38*γ* role in primary human erythroid cells differentiation has been suggested. Analysis of the mRNA expression pattern of each p38 isoform during erythroid differentiation of primary human erythroid progenitors shows that p38*α* and p38*γ* are expressed in early and late stages, whereas p38*δ* mRNA is expressed only at terminal stages of differentiation, indicating a possible role of p38*γ* in hematopoiesis and of p38*δ* during the terminal phase of differentiation [67].

Since p38*γ* expression is very high in skeletal muscle in comparison to other tissues, it is not surprising that it may play a fundamental role in skeletal muscle differentiation. Thus, endogenous p38*γ* protein level increases when myoblast differentiates into myotubes [68, 69]. Moreover, it has been shown that overexpression of p38*γ* in skeletal muscle cells leads to differentiation from myoblast to myotubes and that a dominant-negative mutant of p38*γ* prevented this differentiation process [5]. Recently, studies in p38*γ* null mice reported that p38*γ* plays a cardinal role in blocking the premature differentiation of skeletal muscle stem cells, the satellite cells that participate in adult muscle regeneration. p38*γ* phosphorylates the transcription factor MyoD and promotes MyoD association to the histone methyltransferase KMT1A. This complex acts repressing transcription and the premature expression of myogenin [38]. This is in contrast with the essential role of p38*α* in muscle differentiation [1, 70]. Moreover, p38*γ* is involved in muscle-specific exercise-induced skeletal muscle adaptation, and it seems to be required for the upregulation of PGC-1*α* (peroxisome proliferator-activated receptor-*γ* (PPAR*γ*) coactivator-1*α*) in mitochondrial biogenesis and angiogenesis in response to exercise and nerve stimulation in mice [71].

## 6. Conclusion

Most of the studies to date have focused on the role of the p38*α* isoform and report the implication of this p38MAPK isoform in numerous biological and physiological processes. However, the *in vivo* functions of other alternative p38 isoforms or the molecular mechanism by which these kinases regulate particular cell processes remain largely unknown, and several important questions remain to be answered to address why a variety of p38MAPK isoforms is needed in mammalian cells are: for example, (i) how the p38MAPK isoforms are differentially activated by certain stimuli to mediate specific nonredundant signals, (ii) the identification of specific physiological substrates and how they are modulated by each p38 isoform, and (iii) the elucidation of new *in vivo* roles. The use of a combination of genetically modified mice, such as mouse lacking one or more p38 isoforms, tissue-specific knock-out mice, and knock-in mice expressing inactive p38MAPK will be a powerful tool to elucidate *in vivo* functions. Furthermore, high throughput genomic and proteomic technologies will also help to answer these questions and to generate enough knowledge that hopefully could be translated in therapeutics strategies by targeting the alternative, p38*γ* and p38*δ* MAPK isoforms.
